# Long-term biodegradation of aged saline-alkali oily sludge with the addition of bulking agents and microbial agents

**DOI:** 10.1098/rsos.180418

**Published:** 2018-10-31

**Authors:** Shijie Wang, Xiang Wang

**Affiliations:** 1 Beijing Municipal Research Institute of Environmental Protection, Beijing 100037, People's Republic of China; 2 State Key Laboratory of Coal Mine Disaster Dynamics and Control, Chongqing University, Chongqing 400044, People's Republic of China; 3 Department of Environmental Science, Chongqing University, Chongqing 400044, People's Republic of China

**Keywords:** bioaugmentation, biodegradation, bulking agent, biostimulation, microbial agent, oil sludge

## Abstract

Huge amount of aged oily sludge was generated during the drilling and transportation of crude oil. Sometimes, the sludge exhibited characters of combined pollution, such as saline-alkali oily sludge. Orthogonal experiments of L_16_(4^5^) were conducted to evaluate the long-term effects of total petroleum hydrocarbons (TPH) concentration, microbial agents (Oil Gator and ZL) and bulking agents (peat and wheat bran) on the biodegradation of aged saline-alkali oily sludge. Compared with the control group, the significant improvement in the removal rate of TPH was exhibited with the addition of microbial agents and bulking agents after 231 days of the experimental period. Based on the values of mean range (*R*), it was revealed that the predominant influencing factor of the bioremediation was TPH concentration. After biostimulation and bioaugmentation, the quantity of petroleum hydrocarbon-degrading bacteria in the oily sludge increased by 2–4 orders of magnitude. Furthermore, the bioremediation improved the microbial diversity based on the analysis of PCR-DGGE. It was inferred that the addition of microbial agents and bulking agents reconstructed the microbial ecological niche. The principal component analysis indicated that the differentiation of the microbial community was generated by the biostimulation and bioaugmentation in comparison with the control samples.

## 1. Introduction

Oily sludge is classified as a hazardous waste in China. Its accidental or deliberate releases pose a serious environmental problem. It is estimated that the amount of aged oily sludge is about 18.85 million tons in China. As petroleum contains hazardous chemicals, such as benzene, toluene, ethylbenzene, xylenes and naphthalene [1], it is toxic, mutagenic, carcinogenic and hazardous to the environment and human health [2,3]. The treatment methods, such as natural attenuation, incineration, solvent extraction and landfill, are usually high-cost or time-consuming [4,5]. Bioremediation has recently attracted significant interest as a reliable and relatively cost-effective technology to deal with this type of pollution [6,7].

The oily sludge is generally composed of petroleum, water, emulsions and solid particles. The complex components result in lower porosity, air permeability and water holding capacity, which all showed a negative influence on the biodegradation process [8,9]. Hydrocarbons in the oily sludge were mostly adsorbed on solid particles and colloids, especially on clay particles, organic matter or oil-mineral aggregation [10]. The biodegradation was limited by mass transfer (e.g. hydrocarbons, oxygen, microbial cells and nutrients) [11]. Research showed that the biodegradation of crude oil was also limited by the species of microorganism [12–14].

Many components of petroleum are biodegradable [15,16]. Hydrocarbon-degrading microorganisms are ubiquitous in the environment. The enhanced biodegradation of oily sludge can be achieved by stimulating the microbial activity [17,18]. The techniques include land farming, composting [19–21], slurry-phase bioreactor [22,23] and biopile [24,25]. Higher biodegradation efficiency can be achieved by the inoculation of exogenous microbial agents [8,26]. Furthermore, the bioremediation of oily sludge can be significantly improved with the addition of bulking agents, such as peat, wood chips, hay, sawdust, bark and manure [27,28].

The commercial microbial agents are widely used since the Exxon Valdez incident [29,30]. However, after the addition of microbial agents, the knowledge for the biodegradation process of the oily sludge (especially the aged oily sludge), which is influenced by multiple factors, has not been explored clearly. In this study, the orthogonal experiment was conducted with five factors (the total petroleum hydrocarbons (TPH) concentration, two types of microbial agents and two types of bulking agents) to exhibit: (i) the improvement of biodegradation efficiency with the addition of microbial agents and bulking agents; (ii) the key factors in the process of oil biodegradation based on the orthogonal analysis; (iii) the changes of microbial community and diversity over a long period of bioremediation (231 days).

## 2. Material and methods

### 2.1. The experimental design

The oily sludge was sampled in Shengli oilfield (37°53′14″ N, 119°02′49″ E, East China), which has been weathered in the open air for 20 years. The physico-chemical property of aged oily sludge is shown as electronic supplementary material, table S1, which exhibits that: (i) the initial TPH concentration (22.61%) was too high and may have toxic effect on the microorganisms and (ii) after storing in the open air for 20 years, the percentages of resins and asphaltenes of the oily sludge were up to 8.45% and difficult to biodegrade.

Two commercial microbial agents, Oil Gator and ZL, were applied in the research. Oil Gator (Gator International, Canada) is produced from recycled cotton seeds containing encapsulated microbes, nitrogen and phosphorus; ZL (Tianjin Genius Technology & Engineering Co. Ltd, China), is composed of nutriment, biosurfactant and hydrocarbon-degrading microbes. After activation by the addition of water, the functional microbes can rapidly use the available hydrocarbon as a substrate. The bulking agents of peat (from Jilin province of China) and wheat bran were both inexpensive and easily obtained in China.

According to the orthogonal experimental design method (L_16_(4^5^)) (electronic supplementary material, table S2), 16 trials were conducted for the bioremediation of aged oily sludge. Based on the orthogonal experiment, the treatment of each trial is shown as electronic supplementary material, table S3. The oily sludge, peat and wheat bran were air-dried and sieved through the 2 mm mesh. Then 1000 g of the mixture (oily sludge, microbial agents, peat and wheat bran) was transferred into 16 flasks. During the experiment, all the flasks were placed in the greenhouse (temperature: 25°C; moisture: 30%) with aeration once a week by thoroughly mixing. The TPH was determined on days 0, 14, 28, 49, 105 and 231.

### 2.2. Analysis of total petroleum hydrocarbon

Based on our previous study [31–33], the TPH concentration of aged oily sludge was analysed by accelerated solvent extractor (ASE300, Dionex, USA). About 10 g of oily sludge was extracted with 60 ml of trichloromethane using an accelerated solvent extractor. The extraction was separated from the solvent using a rotary evaporator and measured gravimetrically to calculate the weight percentage of TPH.

### 2.3. Enumeration of petroleum hydrocarbon-degrading bacteria

The quantity of petroleum hydrocarbon-degrading bacteria was assessed by the plate count method. The colony-forming units (CFU) of TPH biodegrading bacteria were determined on days 0, 7, 14, 28, 49, 105 and 231. Ten grams of oily sludge sample was weighed and transferred to a bottle which contained 100 ml of sterile pyrophosphate buffer (0.2%). After shaking at 500 r.p.m. for 30 min and centrifugation at 1500 r.p.m. for 5 min, the suspension was then treated with gradient dilution technique in 10-fold with 0.85% (w/v) NaCl solution. The solidified mineral medium consisted of NaCl (5 g), K_2_HPO_4_ (1 g), NH_4_H_2_PO_4_ (1 g), (NH_4_)_2_SO_4_ (1 g), sterile water (1000 ml) (from Milli-Q system, USA) and agar (20 g). Besides, 0.1% (w/v) of sterilized crude oil served as the sole carbon source. The colony density was recorded after incubation at 25°C for 20 days.

### 2.4. Analysis of microbial community

The last batch of samples was used to analyse the microbial community using the method of polymerase chain reaction denaturing gradient gel electrophoresis (PCR-DGGE). Total genomic DNA was extracted according to the protocol [34,35]. Bacterial 200 bp fragments of the 16S rRNA gene (V3 region) were amplified with the primers (338f, forward: 5′-ACTCCTACGGGAGGCAGCAG-3′; 534r, reversed: 5′-ATTACCGCGGCTGCTGG-3′). PCR was performed using a 25 µl (total volume) mixtures, containing 1 µl of each primer (12.5 pmol), 5 µl of each deoxyribonucleoside triphosphate (200 µmol), 5 µl of 10× PCR buffer (100 mmol Tris–HCl, 15 mmol MgCl_2_, 500 mmol KCl, pH 8.3) and 0.5 µl of Taq DNA polymerase (Promega Co., USA), and made up to 25 µl with sterile water. PCR products were confirmed by 2.0% (w/v) agarose gel with ethidium bromide (0.5 µg l^−1^) staining. Amplified DNA was then purified using a Qiaquick PCR cleanup kit (Qiagen Inc., USA). The PCR-amplified fragments were separated by DGGE using a Dcode universal mutation detection system (BioRad, USA). After electrophoresis, the gel was stained with ethidium bromide for 30 min and photographed with a Gel Doc 2000 gel imaging system (BioRad, USA).

### 2.5. Data analysis

The orthogonal data processing was done through mean range (*R*) analysis. The banding patterns of DGGE profile were analysed by Quantity One (v. 4.5) software (BioRad, USA). The normalized data were subsequently used for principal component analysis (PCA).

## 3. Results and discussion

### 3.1. Biodegradation of the TPH

**Figure 1.**
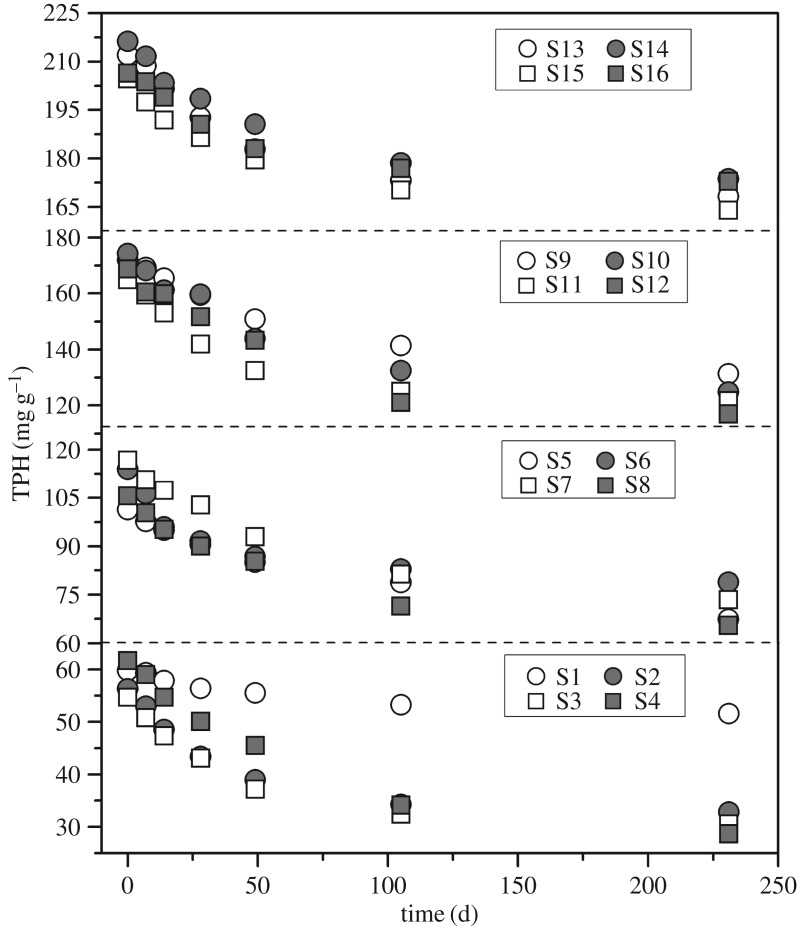
The temporal change of TPH concentration during the experimental period. The 16 trials were classified into four groups based on the initial TPH (58.11 ± 3.19, 109.41 ± 7.14, 169.7 ± 4.05 and 209.84 ± 5.30 mg g^−1^).

The initial four TPH concentration levels of the 16 trials were 58.11 ± 3.19 mg g^−1^ (5% group), 109.41 ± 7.14 mg g^−1^ (10% group), 169.7 ± 4.05 mg g^−1^ (15% group) and 209.84 ± 5.30 mg g^−1^ (20% group), respectively. As shown in figure 1, all the 16 trials exhibited a significant TPH decline over 231 days. Although the 16 trials were at different TPH concentration levels, they all showed a similar two-phase degradation trend: the TPH degradation was at a typical first-order kinetic in the first 105 days, and then levelled off during the 105 to 231 days. The above degradation pattern was assumed to correlate with the change of bioavailability of petroleum hydrocarbons.

The TPH removal rates of 16 trials were in the range from 13.62 to 53.48%. The lowest and highest TPH removal rate appeared in trials 1 and 4, respectively. Among the 16 trials, the trial 1 served as control (at the 5% TPH level) without the addition of bulking and microbial agents. In comparison with the control, a significantly higher removal rate of TPH was observed in other trials, which indicated that the microbial agents and bulking agents showed a positive effect on the elimination of petroleum contaminants. In this study, the average TPH removal rates of the four TPH concentration levels were 38.27 ± 8.78% (5%, S1–S4), 34.85 ± 3.33% (10%, S5–S8), 27.22 ± 2.99% (15%, S9–S12) and 19.11 ± 3.26% (20%, S13–S16), which suggested that the biodegradation of oily sludge might be dependent on the petroleum hydrocarbon concentration.

### 3.2. Evaluation of influencing factors on the biodegradation

The mean range (*R*) of orthogonal analysis was used to study the effect of the five factors (TPH, the microbial agents of Oil Gator and ZL, and the bulking agents of wheat bran and peat) on the biodegradation of petroleum hydrocarbons. It is proved that the biodegradation of petroleum hydrocarbons is a complex process involving mass transfer (sorption, desorption, dissolution and diffusion), biodegradation of petroleum hydrocarbons, microbial growth and reproduction [28,36].

**Figure 2.**
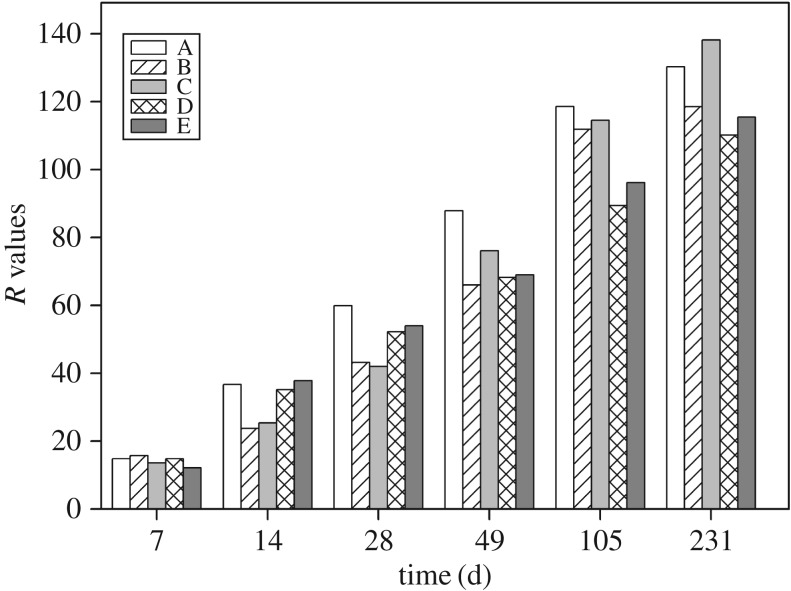
The mean range (*R*) values of influencing factors during the 231 days of bioremediation. The factor A, B, C, D and E stands for the TPH, Oil Gator, ZL, wheat bran and peat, respectively.

As shown in figure 2, the determining factors varied with time. However, the *R* values of TPH concentration in different periods were generally higher than the other four factors. It indicated that the TPH concentration was the predominant determining factor of the bioremediation. The *R* values of the five factors on the seventh day were not significantly different. It might be due to the domestication period for adaption. On the 14th and 28th days, besides the TPH, the peat and wheat bran became the dominant factor. The function of bulking agents is to increase porosity, oxygen diffusion and water holding capacity [27,28], and serve as co-substrate for the biodegradation of complex hydrocarbons [27,37]. The *R* values of Oil Gator and ZL increased gradually during days 49–231, suggesting that the microbial agents played an important role after the adaption of exogenous microorganism. Based on the *R* and cost-effective analysis, the optimal condition combination was A1B3C3D4E4 (detailed information is shown in electronic supplementary material, table S2) for the bioremediation of aged oily sludge.

### 3.3. Characteristics of the microbial quantity and community

**Figure 3.**
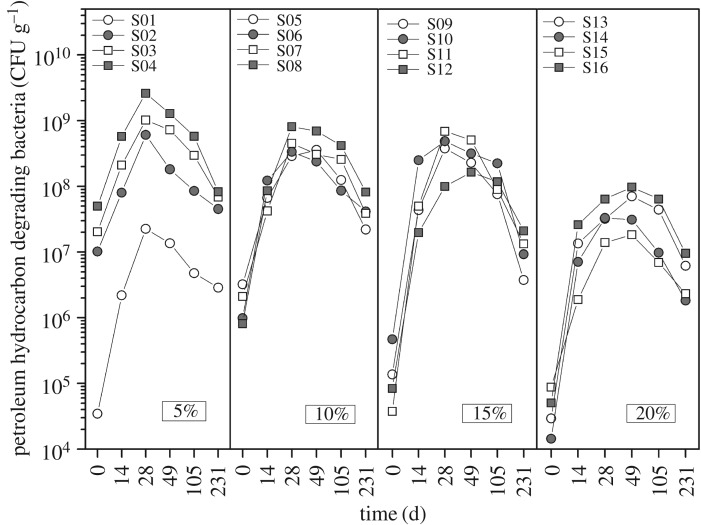
The temporal changes of quantity of the petroleum hydrocarbon-degrading bacteria. S01–S16 stands for the 16 orthogonal trials which are classified into four groups of 5, 10, 15 and 20% according to the initial TPH concentrations.

As shown in figure 3, the TPH concentration had a great effect on the initial quantity of the petroleum hydrocarbon-degrading bacteria. The orders of magnitude were 10^7^, 10^6^, 10^5^ and 10^4^ corresponding to the TPH concentration level group of 5, 10, 15 and 20%. It was observed that the initial quantity of the petroleum hydrocarbon-degrading bacteria of the control (trial 1) was only on the level of 10^4^. It indicated that, without bioaugmentation and biostimulation, the toxicity of the petroleum hydrocarbons resulted in relative low biomass of indigenous microbes. As shown in figure 3, the numbers of the bacteria increased by two to four orders of magnitude during 28–49 days. At the same time, the TPH removal rate was at a high level. The correlation suggested that the biodegradation of petroleum hydrocarbons was determined by the microbial population in a way [38]. However, the bacteria counts decreased to 10^6^–10^8^ CFU g^−1^ during the 49–231 days. It might be due to the low bioavailability of the residual petroleum materials.

**Figure 4.**
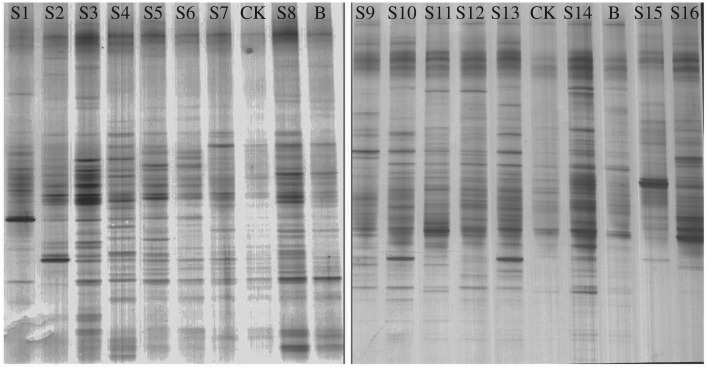
DGGE profiles of the last batch of samples after 231 days of bioremediation. The lane labelled with S1–S16, B and CK stands for the samples of 16 trials, background soil and pure oily sludge, respectively.

The PCR-DGGE profile is shown in figure 4. Distinctly visible bands were hardly detected in the CK profile (pure aged oily sludge). The differences of visible bands revealed the selectivity of hydrocarbons degrading microbes which varied with different trials. Compared with the CK and background soil (B) which was not contaminated by petroleum hydrocarbons, more visible bands were detected on the profiles of the 16 samples. It suggested that the bioremediation improved the microbial diversity, which might be caused by the microbial agents, bulking agents and lower TPH concentration [39].

**Figure 5.**
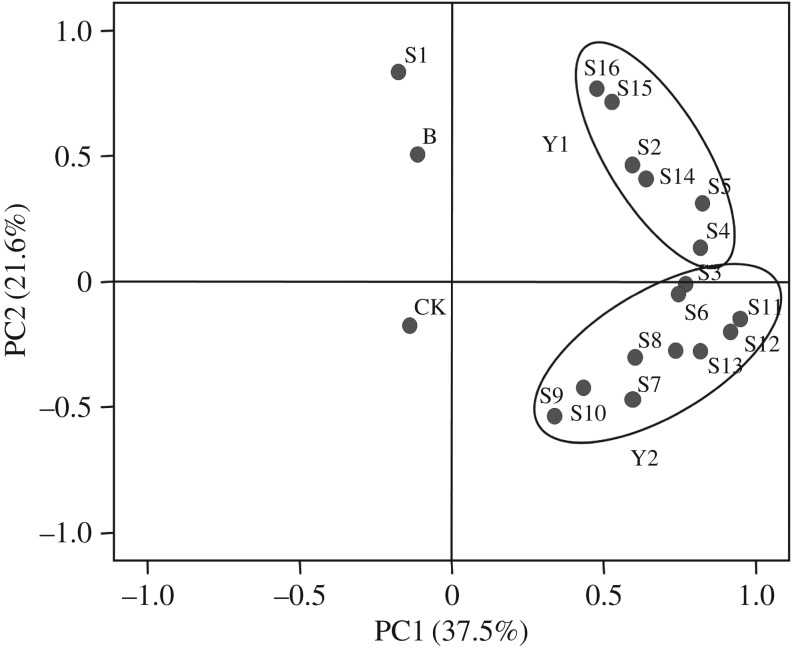
Principal components analysis based on the DGGE fingerprints. The two major clusters are labelled with Y1 and Y2; CK: pure aged oily sludge; B: background soil.

The similarity of the microbial community was analysed using the method of PCA. As shown in figure 5, the percentage of variance explained by PC1 was 37.5%, while PC2 explained 21.7% of the variance. The results of PCA analysis showed that the sample spots were grouped into two major clusters: Y1 (containing the trials S2, S4, S5, S14, S15 and S16) and Y2 (containing the trials S3, S6, S7, S8, S9, S10, S11, S12 and S13). And along the horizontal direction (PC1), Y1 and Y2 are located to the right of the vertical axis, whereas the CK, B and S1 are located to the left. The differentiation might be caused by the addition of microbial agents (Oil Gator and ZL) and bulking agents, which indicated the difference in microbial community.

## 4. Conclusion

Bioremediation with amendments (microbial agents and bulking agents) is an effective approach for the treatment of oily sludge. A significant removal rate of petroleum hydrocarbons was observed over 231 days of bioremediation. The results of the orthogonal experiment (L_16_(4^5^)) demonstrated that the determining factor for the biodegradation of petroleum hydrocarbons was the TPH concentration followed by the addition of bulking agents and microbial agents. The bioremediation improved the microbial diversity based on the analysis of PCR-DGGE.

## Supplementary Material

Table S1

## Supplementary Material

Table S2

## Supplementary Material

Table S3

## Data accessibility

This article does not contain any additional data.
