# Supplementary material for: Long-term biodegradation of aged saline-alkali oily sludge with the addition of bulking agents and microbial agents
Source: R Soc Open Sci. 2018 Oct 31;5(10):180418. doi: 10.1098/rsos.180418 (PMC6227984; doi:10.1098/rsos.180418)
Supplement: Table S1 [file rsos180418supp1.pdf]

Table S1 Physiochemical properties of aged oily sludge

pH	Soil density (kg m^{-3})	Organic matter (w/w)	Salinity (g kg^{-1})	CEC (cmol kg^{-1})	Water content (w/w)	TN (mg kg^{-1})	TP (mg kg^{-1})	TK (mg kg^{-1})
8.60	2.74	2.33%	63.04	5.44	23%	15.00	78.00	1690.00

Soil type	Clay (<0.002mm, w/w)	Fine silt (0.02-0.002mm, w/w)	Sand (2-0.02mm, w/w)
loamy sand	13.71%	29.62%	56.67%

TPH (w/w)	Saturates (w/w)	Aromatics (w/w)	Resins (w/w)	Asphaltenes (w/w)
22.61%	6.98%	7.18%	6.05%	2.40%

As (mg kg^{-1})	Hg (mg kg^{-1})	Cu (mg kg^{-1})	Cd (mg kg^{-1})	Cr (mg kg^{-1})	Zn (mg kg^{-1})	Pb (mg kg^{-1})
8.8	0.02	17.5	0.054	42.3	64.7	26.2
