# Supplementary material for: Long-term biodegradation of aged saline-alkali oily sludge with the addition of bulking agents and microbial agents
Source: R Soc Open Sci. 2018 Oct 31;5(10):180418. doi: 10.1098/rsos.180418 (PMC6227984; doi:10.1098/rsos.180418)
Supplement: Table S2 [file rsos180418supp2.pdf]

**Table S2** Orthogonal experiment design of $L_{16}$ $(4^5)$

| Influencing Factors | Levels (w/w, %) | | | |
|---|---|---|---|---|
| | 1 | 2 | 3 | 4 |
| A: TPH[a] | 5% | 10% | 15% | 20% |
| B: Oil-gator[b] | 0 | 1% | 5% | 10% |
| C: ZL[b] | 0 | 1% | 5% | 10% |
| D: Wheat bran[c] | 0 | 1% | 5% | 10% |
| E: Peat[c] | 0 | 1% | 5% | 10% |

a: mixing oily sludge and clean soil together to decrease the TPH;

b: The mixing ratio of microbial agents to the gross mass;

c: The mixing ratio of bulking agents to the gross mass.
