# Supplementary material for: Long-term biodegradation of aged saline-alkali oily sludge with the addition of bulking agents and microbial agents
Source: R Soc Open Sci. 2018 Oct 31;5(10):180418. doi: 10.1098/rsos.180418 (PMC6227984; doi:10.1098/rsos.180418)
Supplement: Table S3 [file rsos180418supp3.pdf]

Table S3 The treatment based on the orthogonal experiment design of $L_{16}(4^5)^a$

Trial NO.	TPH (w/w, %)	Oil Gator (w/w, %)	Zl (w/w, %)	Wheat Bran (w/w, %)	Peat (w/w, %)
S1	10	1	1	1	0
S2	10	10	5	0	5
S3	5	1	5	10	1
S4	15	5	5	5	0
S5	5	0	0	0	0
S6	5	10	1	5	10
S7	15	0	1	10	5
S8	5	5	10	1	5
S9	20	5	1	0	1
S10	10	5	0	10	10
S11	15	10	0	1	1
S12	10	0	10	5	1
S13	20	0	5	1	10
S14	15	1	10	0	10
S15	20	10	10	10	0
S16	20	1	0	5	5

a: The above table is designed based on the SPSS Statistics 22.
